# Pharmacokinetics, mass balance, and metabolism of [^14^C]SPH3127 (sitokiren), a novel direct renin inhibitor in humans

**DOI:** 10.3389/fphar.2026.1893643

**Published:** 2026-09-01

**Authors:** Sheng Ma, Xintong Huang, Shu Yan, Haonan Liu, Hua Zhang, Yifan Zhang, Weiwei Cui, Cungang Ding, Xingxing Diao, Liyan Miao

**Affiliations:** 1 Department of Pharmacy, the First Affiliated Hospital of Soochow University, Suzhou, China; 2 Institute for Interdisciplinary Drug Research and Translational Sciences, Soochow University, Suzhou, China; 3 Shanghai Institute of Materia Medica, Chinese Academy of Sciences, Shanghai, China; 4 University of Chinese Academy of Sciences, Beijing, China; 5 Tianjin University of Traditional Chinese Medicine, Tianjin, China; 6 Shanghai Pharmaceuticals Holding Co., Ltd., Shanghai, China

**Keywords:** [^14^C]SPH3127, drug metabolism, mass balance, pharmacokinetics, radioactivity, renin inhibitor

## Abstract

**Introduction:**

SPH3127 (Sitokiren), a novel direct renin inhibitor used to treat hypertension, is currently approved for marketing in China. The primary objective of this study was to determine the pharmacokinetics, mass balance, and biotransformation of [^14^C]SPH3127 in humans after the drug was administered to healthy Chinese male subjects.

**Methods:**

The absorption, metabolism, and excretion of SPH3127 were characterised via isotope labeling technology in six healthy Chinese male subjects after receiving a single 100 mg oral dose of [^14^C]SPH3127 (100 μCi).

**Results:**

SPH3127 was rapidly absorbed, with median T_max_ values of 0.417 and 0.75 h observed for the parent drug and total radioactivity in human plasma. The arithmetic mean plasma half-life (t_1/2_) was approximately 4.83 h for SPH3127-related material. After 192 h of dosing, the mean cumulative excreted radioactivity was 101.66% of the dose, with 44.35% in urine and 57.31% in feces, indicating that urinary and fecal excretions were comparable and served as the primary elimination routes. Metabolite profiling revealed that the parent drug was detected in plasma, as well as in feces and urine. Twelve major metabolites in plasma, urine, and feces were analysed and identified. The principal metabolic pathways involved oxidation, dehydrogenation, hydrolysis, glucuronidation, and sulfation. SPH3127 was found to be safe, with no serious adverse events reported.

**Conclusion:**

SPH3127 showed favourable pharmacokinetic characteristics and safety profiles in this study. SPH3127 was rapidly absorbed and extensively metabolized in humans. The main excretion route of SPH3127 and its metabolites was through urine and feces.

**Clinical Trial Registration:**

http://www.chinadrugtrials.org.cn/, identifier CTR20222187.

## 1 Introduction

Hypertension is a common chronic disease and one of the leading causes of cardiovascular disease and death. The aetiology and progression mechanisms of hypertension are highly complex. Various factors, including high-sodium and low-potassium diets, obesity, inflammation, activation of the neuroendocrine system, and genetic alterations, can contribute to the development of hypertension (Harrison et al., 2021). The renin-angiotensin-aldosterone system (RAAS) primarily regulates blood pressure by influencing arterial constriction and intracellular sodium-water retention. Normal RAAS activity is essential for maintaining proper fluid balance and controlling blood pressure. RAAS overactivity is a major contributor to hypertension. Therefore, blocking RAAS represents a critical therapeutic approach for treating hypertension and reducing target organ damage (Azizi and Ménard, 2004).

RAAS inhibitors play a dominant role in antihypertensive therapy. Currently, the primary RAAS system inhibitors used clinically include angiotensin-converting enzyme inhibitors (ACEIs), angiotensin receptor blockers (ARBs), and direct renin inhibitors. However, neither ACEIs nor ARBs can completely block the RAAS system. Long-term use of ACEIs gradually elevates angiotensin (Ang) 2 levels, a phenomenon known as Ang 2 escape. Initial treatment with ARBs causes aldosterone levels to decrease, but some patients experience aldosterone breakthrough after prolonged use (Zhang et al., 2020). Therefore, direct renin inhibitors exhibit superior antihypertensive effects.

**FIGURE 1:**
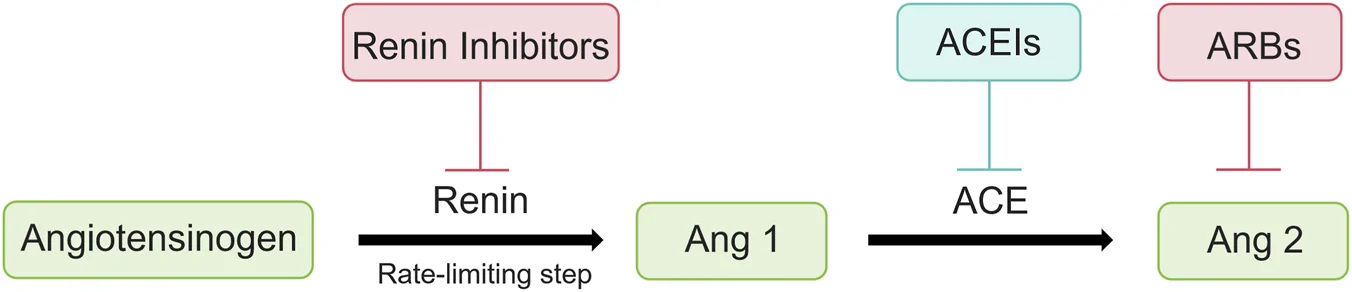
The RAAS pathway and the mechanisms of action of the major therapeutic classes.

Approximately 70% of hypertension cases stem from elevated renin levels (Pool, 2007). Renin, primarily released by juxtaglomerular cells, acts as the rate-limiting enzyme. It catalyses the conversion of angiotensinogen into Ang 1, which is subsequently cleaved by angiotensin-converting enzyme to form Ang 2. Ang 2 can increase blood pressure by constricting blood vessels (Wu et al., 2018; Liu W. et al., 2025). Renin inhibitors can halt the RAAS cascade and lower blood pressure by reducing upstream Ang 1 production (Figure 1). Thus, drugs that directly inhibit renin are preferred options for blocking downstream RAAS pathways.

The first direct renin inhibitor, aliskiren, is FDA-approved as an effective agent blocking the RAAS pathway at its rate-limiting step (Wang et al., 2024; Iijima et al., 2022), with the *t*_1/2_ of 50.1 and 44 h in healthy subjects and patients with diabetes, respectively (Vaidyanathan et al., 2007; Zhao, et al., 2006). It can be used alone or in combination with other drugs to treat hypertension (Oparil et al., 2007; Lanier et al., 2014; Zion and Izzo, 2011). Aliskiren can effectively lower blood pressure within 24 h (Wood et al., 2003). However, several issues remain unresolved. Serious adverse effects have been reported in clinical trials, such as the risk of hyperkalemia (Parving et al., 2012; Harel et al., 2012).

SPH3127 (Sitokiren) is a novel oral non-peptide renin inhibitor developed by Shanghai Pharmaceuticals Holding Co., Ltd (SPH). SPH3127 has been approved for marketing in China on 9 December 2025. SPH3127 has a mechanism of action similar to that of aliskiren, but its chemical structure differs from that of aliskiren. Based on *in vitro* assays using recombinant human renin and human serum renin, SPH3127 exhibits higher reninase inhibitory activity than aliskiren, while showing no inhibitory activity against other aspartic proteases (HIV-1, Pepsin, Cathepsin D, Cathepsin E) (Mao et al., 2019). This specificity may reduce SPH3127s side effects. Furthermore, SPH3127 demonstrates higher bioavailability than aliskiren (Iijima et al., 2022; Vaidyanathan et al., 2008; Jing et al., 2021).

A better understanding of SPH3127 metabolism is crucial to identify its metabolic profile, assess the safety of its metabolites, understand its pharmacokinetics, and determine its mechanisms of clearance. These findings are essential for designing potential drug-drug interaction (DDI), which can guide future clinical studies and help select appropriate dosing strategies (He et al., 2023; Zheng et al., 2022). Additionally, the use of radioactive tracers, such as ^14^C-labeled compounds, in absorption, distribution, metabolism, and excretion studies enables the determination of the mass balance and major metabolic routes of a drug (Zheng et al., 2021; Gao et al., 2025; Ge et al., 2023; Penner et al., 2012; Zheng et al., 2025; Bian et al., 2021; Liu X. et al., 2025).

Therefore, in this study, [^14^C]SPH3127 suspension solution was given to six healthy Chinese male subjects to study the pharmacokinetics, mass balance, and biotransformation of SPH3127.

## 2 Materials and methods

### 2.1 Chemicals and reagents

**FIGURE 2:**
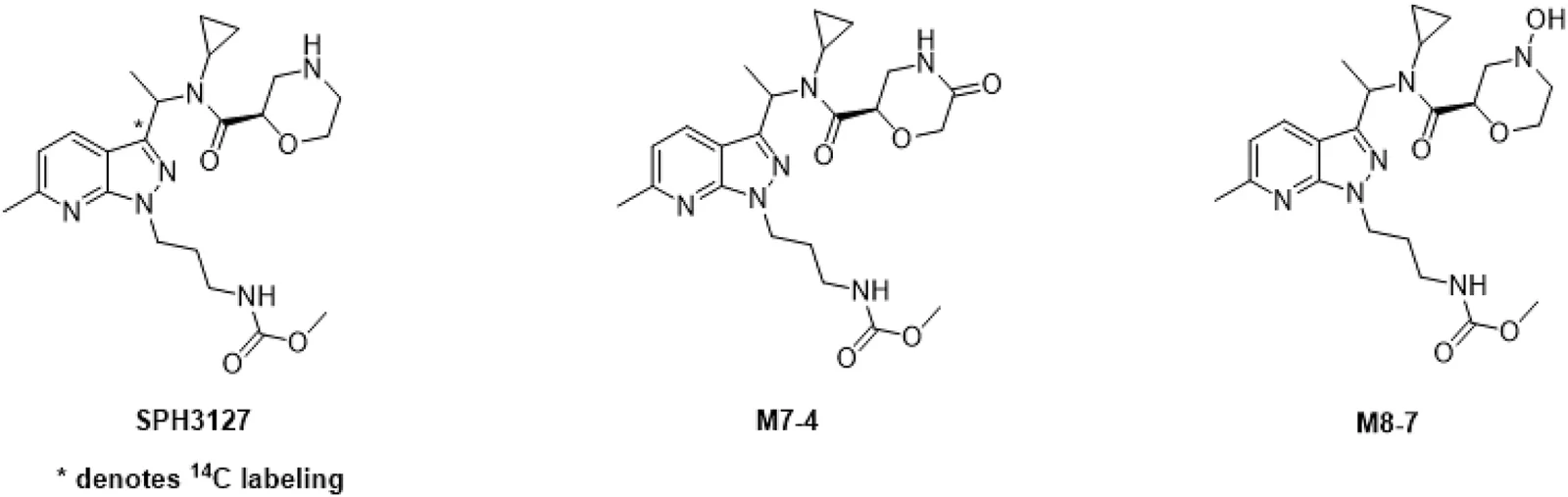
Chemical structures of SPH3127, M7-4 and M8-7 (* indicates ^14^C-labeled position).

SPH3127 tablets were provided by Shanghai Sine Pharmaceutical Laboratories Co., Ltd. (Shanghai, China). [^14^C]SPH3127 (99.80% purity, 53.13 mCi/mmol) was synthesised by Changsha Beta Pharmaceutical Technology Co., Ltd. (Changsha, China). Reference substances of SPH3127 (99.6% purity) and its metabolites M7-4 (93.68% purity) and M8-7 (95.38% purity) were kindly supplied by Shanghai Pharmaceuticals Holding Co., Ltd. (Shanghai, China) (Figure 2).

Liquid scintillation cocktail was purchased from Zhejiang Dingxin Tech (Shaoxing, China). Alkaline liquid scintillation cocktail was purchased from RDC™ BioTech Crop (Hillsdale, NJ, United States). High-performance liquid chromatography (HPLC)-grade methanol was purchased from Sigma (St. Louis, MO, United States). HPLC-grade acetonitrile was purchased from CINC (Shanghai, China). Ammonium acetate and dimethyl sulfoxide (DMSO) were purchased from Sinopharm Chemical Reagent Co., Ltd. (Shanghai, China). Formic acid was purchased from Rhawn Chemical Reagent Co., Ltd. (Shanghai, China). Ultrapure water was generated by a Milli-Q integral water purification system (Millipore, Molsheim, France). The other commercially available reagents used in this study were of analytical grade.

### 2.2 Instruments and conditions

In this study, high-resolution mass spectrometry (HR-MS) and HR-MS^2^ acquisition with mass defect filter and background subtraction techniques were extensively employed for metabolite identification. HR-MS and HR-MS^2^ acquisitions were performed using a Vanquish ultra-high-performance liquid chromatography (UHPLC) system coupled with a Q Exactive Plus mass spectrometer (Thermo, Waltham, MA, United States) equipped with an electrospray ionisation (ESI) source. Chromatographic separation was achieved on an Agilent Extend C18 column (150 mm × 4.6 mm, 5 μm) with a Phenomenex C18 guard column (10 mm × 4.6 mm, 5 μm) at 25 °C with a flow rate of 0.6 mL/min. The mobile phase consisted of 5 mM ammonium acetate in water with 0.1% formic acid (A) and acetonitrile (B). The gradient elution program was as follows: 0 min, 5% B; 2 min, 5% B; 45 min, 35% B; 45.5 min, 95% B; 48 min, 95% B; 49 min, 5% B; 60 min, 5% B. The eluent was monitored by ultraviolet (UV) detection at a wavelength of 190–500 nm. The mass spectrometer was operated in positive ESI mode with a scan range of m/z 80–1,200 Da. The MS parameters were as follows: sheath gas, 35 L/min; aux gas, 10 L/min; capillary temperature, 320 °C; capillary voltage, 3.8 kV; and collision energy, 10, 20, and 30. Data were acquired using Xcalibur software (Thermo) and analysed using Compound Discoverer software (Thermo).

### 2.3 Study design, subjects, and sample collection

This open-label, single-center, and single-dose study was conducted at the First Affiliated Hospital of Soochow University (Suzhou, China). Six healthy Chinese males aged 20–30 years with a body mass index of 19.6–24.5 kg/m^2^ were included. This study was conducted in accordance with the ethical principles outlined in the Declaration of Helsinki (ClinicalTrials.gov ID: NCT05593562) and was approved by the Hospital Ethics Committee (Approval No. 2022-172). All participants signed a written informed consent form before commencing this study. The subjects were discharged from the clinic when the following criteria were met: the accumulated excreted radioactivity was more than 80% of the administered radioactivity, the excreted radioactivity was lower than 1% of the administered radioactivity over two consecutive days, and the measured plasma radioactivity concentration was less than threefold that of predose. Each of the six subjects received a single oral dose of 100 mg [^14^C]SPH3127 (100 µCi) as a suspension in approximately 240 mL of water. Subjects were fasted for at least 10 h before receiving SPH3127 and remained fasted for 4 h after receiving the drug. No fluids were allowed from 1 h before and after receiving SPH3127.

Plasma samples were collected from predose to 96 h after dose. Blood samples were collected from predose to 24 h after dose. Urine and feces samples were collected from predose to 192 h after dose administration.

#### 2.3.1 Plasma and blood

Plasma samples were collected 1 h before dosing and 0.17, 0.33, 0.5, 0.75, 1, 1.5, 2, 4, 8, 12, 24, 48, 72, and 96 h post-dosing. Approximately 8 mL of venous blood was collected at each time point using K_2_EDTA blood collection tubes for anticoagulation. Plasma was separated by centrifugation for nonradioactive and radioactive pharmacokinetic studies. Additionally, about 8 mL of venous blood was collected 1 h before dosing, and 0.5, 1, 2, 4, 8, 12, and 24 h post-dosing, 2 mL of which was set aside for total radioactivity assessment in blood, and the remaining was centrifuged to isolate plasma for metabolic radio-profiling and metabolite identification.

#### 2.3.2 Urine

Urine samples were collected at predose and 0–4, 4–8, 8–24, 24–48, 48–72, 72–96, 96–120, 120–144, 144–168, and 168–192 h postdose.

#### 2.3.3 Feces

Fecal samples were collected at predose and 0–24, 24–48, 48–72, 72–96, 96–120, 120–144, 144–168, and 168–192 h postdose.

Blood and plasma samples were stored at −70 °C until assay, while urine and fecal samples were stored at −20 °C.

### 2.4 Safety assessment

Safety evaluation indicators include adverse events (AEs), serious adverse events (SAEs), laboratory tests (hematology, serum chemistry, coagulation function, urinalysis, fecal occult blood), vital signs, 12-lead electrocardiogram, and physical examination. All AEs and SAEs were monitored and graded according to Common Terminology Criteria for Adverse Events (CTCAE) Version 5.0.

### 2.5 Total radioactivity analysis

Total radioactivity in plasma and urine was measured using a liquid scintillation counter (LSC, Hidex, Turku, Finland). Each fecal sample was mixed with twice its weight of methanol-water (1:1, *v*/*v*) and homogenized. Blood and fecal homogenates were aliquoted and combusted using an OX-501 Biological Oxidizer (Harvey, Hillsdale, United States). Generated ^14^CO_2_ was then trapped in alkaline RDC liquid scintillation cocktail (Hillsdale, NJ, United States) and measured using LSC. The combustion efficiency of the oxidizer was determined by burning a standard solution containing a known amount of [^14^C] radioactivity. The LSC was programmed to automatically subtract the instrument background (including cocktail) and convert counts per minute (CPM) to disintegrations per minute (DPM).

### 2.6 Sample preparation for metabolic radioprofiling

#### 2.6.1 Plasma

The plasma samples from six subjects were collected at 0 h before dosing and at 0.17, 0.33, 0.5, 0.75, 1, 1.5, 2, 4, and 8 h after dosing, and were pooled according to the AUC-pooling principle (Hop et al., 1998). From each subject, 0.017 mL of plasma was pooled at 0 h, 0.033 mL at 0.17 h, 0.033 mL at 0.33 h, 0.042 mL at 0.5 h, 0.05 mL at 0.75 h, 0.075 mL at 1 h, 0.1 mL at 1.5 h, 0.25 mL at 2 h, 0.6 mL at 4 h, and 0.4 mL at 8 h, resulting in a total of 1.6 mL of samples. The plasma samples from the six subjects were then mixed in equal volumes to produce a single plasma sample with a volume of 9.6 mL. An aliquot of the pooled plasma sample was extracted by adding two volumes of methanol-acetonitrile (50:50, *v*/*v*). The mixture was subsequently vortexed for 1 min and sonicated for 1 min twice before centrifugation (5,000 rpm, 10 min). The supernatant was transferred into another clean tube, and then 0.2 volume of water was added to the post-extraction solid. The mixture was vortexed for 1 min and sonicated for 1 min. Then, one volume of methanol-acetonitrile (50:50, *v*/*v*) was added to the above mixture, which was vortexed for 1 min and sonicated for 1 min twice before centrifugation (5,000 rpm, 10 min). The two supernatants were combined, and the extraction recovery was measured. The supernatant was dried under nitrogen. The residues were reconstituted with an appropriate volume of acetonitrile-water (20:80, *v*/*v*), which was then injected into the UHPLC-Fraction collector system (Thermo). The UHPLC eluent was collected in Deepwell LumaPlate 96-well plates (PerkinElmer) for 0–60 min at 10 s intervals. After evaporation, the radioactivity was determined with an offline Sense Beta plate reader (Hidex, Finland). Data were reconstructed using the Laura software (Lablogic, Sheffield, United Kingdom) to obtain a radio-chromatogram. A portion of the reconstituted solution was injected for UHPLC-Q Exactive Plus MS analysis.

#### 2.6.2 Urine

According to the cumulative radioactivity measured from 0–192 h post-dose, radioactivity in the 0–24 h urine samples accounted for more than 90% of total urine radioactivity. An equal percentage (based on volume) of each urine sample was pooled for 0–24 h from the six subjects. The pooled urine sample was centrifuged (5,000 rpm, 10 min), and the recovery was measured after centrifugation. The supernatant was dried under nitrogen. The residues were reconstituted in acetonitrile-water (20:80, *v*/*v*), and a portion of which was injected for UHPLC-β-RAM (Lablogic, Sheffield, United Kingdom) online radioactivity detection. Another portion of the reconstituted solution was injected for UHPLC-Q Exactive Plus MS analysis.

#### 2.6.3 Feces

According to the cumulative radioactivity measured for 0–192 h post-dose, 0–72 h fecal radioactivity accounted for more than 90% of the total fecal radioactivity. An equal percentage (based on weight) of each fecal homogenate sample was pooled for 0–72 h across the six subjects. An aliquot of pooled fecal homogenate was extracted by adding three volumes of methanol-acetonitrile (50:50, *v*/*v*). The mixture was subsequently vortexed for 1 min and sonicated for 10 min twice before centrifugation (5,000 rpm, 10 min). The supernatant was transferred into another clean tube, and then 0.5 volume of water was added to the post-extraction solid. The mixture was vortexed for 1 min and sonicated for 10 min. Then, one volume of methanol-acetonitrile (50:50, *v*/*v*) was added to the above mixture, which was vortexed for 1 min and sonicated for 10 min twice before centrifugation (5,000 rpm, 10 min). The two supernatants were combined, and the extraction recovery was measured. The supernatant was dried under nitrogen. The residues were reconstituted with an appropriate volume of acetonitrile-water (20:80, *v*/*v*), and a portion of which was injected for UHPLC-β-RAM (Lablogic, Sheffield, United Kingdom) online radioactivity detection. Another portion of the reconstituted solution was injected for UHPLC-Q Exactive Plus MS analysis.

### 2.7 Metabolite identification

The structures of metabolites were identified by UHPLC-Q Exactive Plus MS by comparing their mass spectral fragmentations with those produced by the parent compound (SPH3127) and other reference substances (M7-4 and M8-7). The molecular ion peaks corresponding to the prominent radioactive chromatographic peaks were analyzed. The software applied for drawing chemical structures is ChemDraw software (version 16.0; PerkinElmer, Norwalk, United States).

### 2.8 Determination of SPH3127, M7-4, and M8-7 in human plasma

The plasma sample was obtained to determine the concentration of SPH3127, M7-4, and M8-7. The analytes and IS (SPH3127-D3) were separated on a Venusil ASB C18 column (50 mm × 4.6 mm, 5 μm; Agela Technologies, Tianjin, China) with temperature maintained at 40 °C. The mobile phase consisted of water with 0.2% FA and 5 mM ammonium acetate and methanol-acetonitrile (1:2, *v*/*v*) with 0.1% FA at a flow rate of 0.6 mL/min. SPH3127, M7-4, and M8-7 were quantitated using a 6,500 triple quadrupole mass spectrometer (Sciex, MA, United States) coupled with an LC-30AD HPLC system (Shimadzu, Kyoto, Japan). The quantitative transition ion pairs were *m/z* 445.2→275.3 (SPH3127), *m/z* 459.2→275.0 (M7-4), *m/z* 461.2→275.1 (M8-7) and *m/z* 448.3→275.2 (IS). Analyst 1.7.2 software (Sciex) was employed to acquire and process data. The calibration curves were linear over the plasma concentration ranges of 1.00–1000 ng/mL for SPH3127, 2.00–2000 ng/mL for M7-4, and 0.40–400 ng/mL for M8-7. The lower limits of quantification for SPH3127, M7-4, and M8-7 were 1.00, 2.00, and 0.40 ng/mL, respectively. The accuracy and precision data are shown in Supplementary Table S1. For the selectivity of SPH3127, the chromatographic peak areas at the retention time of SPH3127 measured in blank plasma from six different sources, hemolytic plasma from one source, and lipemic plasma from one source were all less than 1.5% of the peak area in the LLOQ sample; for M7-4, they were less than 2.0%; and for M8-7, they were less than 12.4%. The peak areas of IS (SPH3127-D3) were less than 0.1% of that of LLOQ sample. The selectivity met the acceptance criteria. For recovery and matrix effect, the mean extraction recoveries of SPH3127 were 107.5%, 102.1%, and 99.6% at concentrations of 3.00, 50.0, and 800 ng/mL, respectively. The recoveries of M7-4 were 105.6%, 101.5%, and 98.3% at concentrations of 6.00, 100, and 1,600 ng/mL, respectively. The recoveries of M8-7 were 101.5%, 102.6%, and 97.8% at concentrations of 1.20, 20.0, and 320 ng/mL, respectively. The recovery of SPH3127-D3 at a concentration of 50.0 ng/mL was 105.7%. In blank plasma, the internal standard-normalized matrix effect factors for SPH3127 at 3.0 and 800 ng/mL were 101.6% ± 3.5% and 101.6% ± 1.8%, respectively. For M7-4, the internal standard-normalized matrix effect factors at 6.0 and 1,600 ng/mL were 97.1% ± 3.1% and 102.2% ± 1.9%, respectively. For M8-7, the internal standard-normalized matrix effect factors at 1.2 and 320 ng/mL were 100.9% ± 8.7% and 101.6% ± 1.8%, respectively. The matrix effects of SPH3127, M7-4, and M8-7 in hemolytic plasma and lipemic plasma are shown in Supplementary Table S2. The matrix effect could be ignored under the present experimental conditions. SPH3127, M7-4, and M8-7 were stable under these study conditions (Unpublished data).

### 2.9 Pharmacokinetics analysis

The plasma concentration-time profiles for SPH3127 and its metabolites, as well as the total radioactivity concentration-time profiles for [^14^C]SPH3127-related components in plasma and blood, were evaluated using non-compartmental methods with Phoenix WinNonlin software (version 7.0; Certara, Mountain View, CA, United States). The methodology used for calculating AUC was the linear-up/log-down trapezoidal method.

The following parameters were calculated: area under the curve (AUC) for plasma concentration versus time up to infinity ( ${\text{AUC}}_{0-\infty }$ ), maximum plasma concentration (C_max_), time to reach C_max_ (T_max_), and half-life (t_1/2_). The urinary and fecal excretion of radioactivity, expressed as a percentage of the administered dose, was determined by dividing the amount of excreted radioactivity by the total administered radioactivity.

## 3 Results

### 3.1 Safety assessment

Three of six volunteers (50.0%) experienced six AEs in this study. The reported AEs were elevated serum uric acid (3, 50.0%), elevated alanine aminotransferase (1, 16.7%), and elevated serum triglycerides (1, 16.7%) with Grade 1 or 2, and vasovagal response (1, 16.7%) with Grade 3. Subjects recovered without any medical treatment by the end of the study. All AEs except the vasovagal response were evaluated to be drug-related. No serious AEs (SAEs) were reported in this study. No AEs leading to subject withdrawal and no subject deaths occurred.

### 3.2 Pharmacokinetics

**FIGURE 3:**
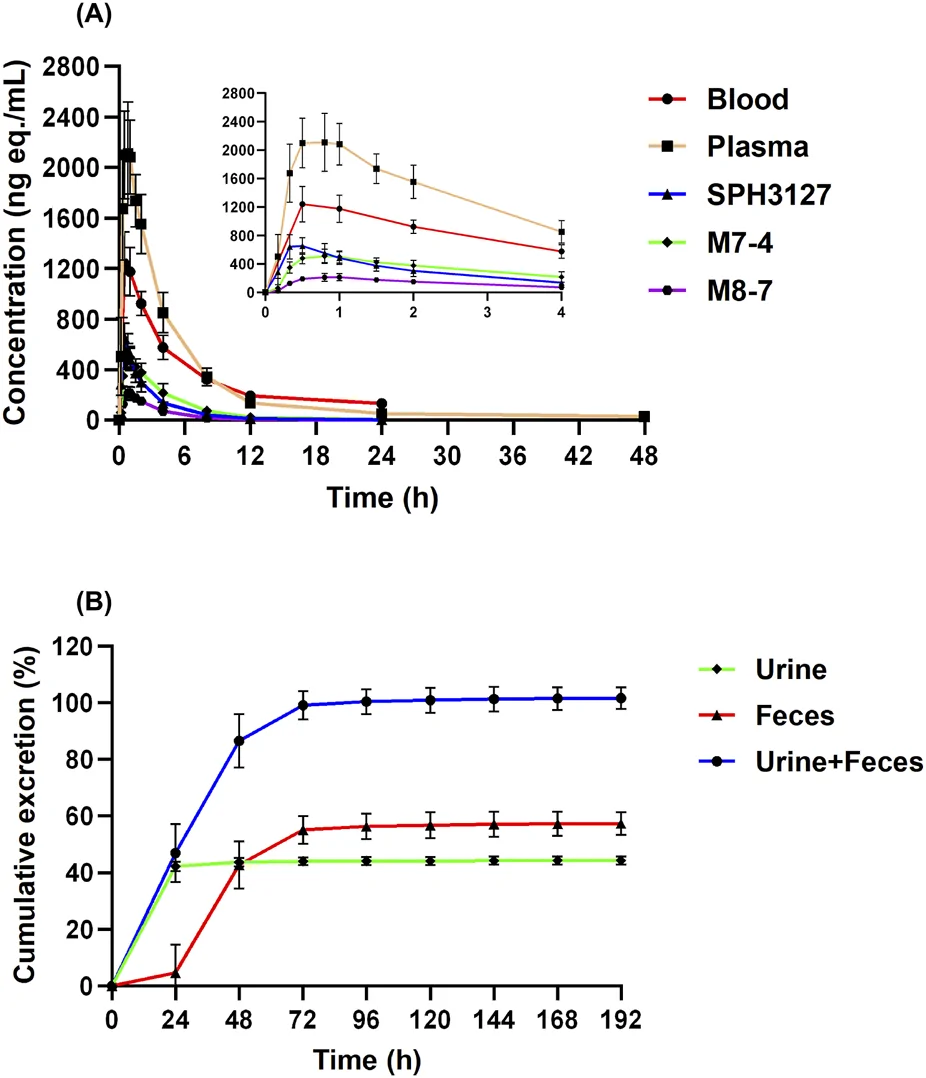
Pharmacokinetics and mass balance of SPH3127 following a single oral administration of 100 mg (100 μCi) [^14^C]SPH3127. **(A)** The concentration-time curve of [^14^C]SPH3127-derived radioactivity in human blood and plasma, and SPH3127, M7-4, and M8-7 in human plasma. **(B)** Total cumulative radioactive dose recovered in urine and feces (%).

The total radioactivity concentration-time profiles in the blood and plasma following a single oral administration of 100 mg [^14^C]SPH3127 (100 µCi) to healthy Chinese male subjects are depicted in Figure 3A, and the associated pharmacokinetics (PK) parameters are summarized in Table 1. The arithmetic mean C_max_ and ${\text{AUC}}_{0-\infty }$ of total radioactivity were 2240 ng eq./mL and 10,300 ng eq./mL·h in plasma, respectively. The median T_max_ and arithmetic mean t_1/2_ in plasma were approximately 0.75 h and 4.83 h, respectively. The ratio of mean total drug-related substance concentration in whole blood to that in plasma (B/P_AUC_) was 0.94, suggesting a near-equal distribution of the drug between plasma and blood cellular components.

**TABLE 1:** Pharmacokinetics parameters of SPH3127 and drug-related components in blood and plasma after a single oral administration of [^14^C]SPH3127 suspension to six healthy Chinese male volunteers (means ± SD), as analysed using noncompartmental methods with Phoenix WinNonlin.

Parameter	SPH3127 (N = 6)		M7-4 (N = 6)	M8-7 (N = 6)	[^14^C]SPH3127-related components (N = 6)		
	Unit	Plasma	Plasma	Plasma	Unit	Plasma	Blood
*C*_max_	ng/mL	691 (139)	526 (84.1)	219 (50.3)	ng eq./mL	2,240 (303)	1,260 (212)
*C*_max__D	ng/mL/mg	6.91 (1.39)	—	—	ng eq./mL/mg	22.4 (3.03)	12.6 (2.12)
AUC_0-t_	ng/mL·h	1780 (406)	2,190 (566)	761 (197)	ng eq./mL·h	9,890 (1970)	7,800 (1,290)
AUC_0−t__D	ng/mL/mg·h	17.8 (4.06)	—	—	ng eq./mL/mg·h	98.9 (19.7)	78.0 (12.9)
${\text{AUC}}_{0-\infty }$	ng/mL·h	1790 (411)	2,220 (569)	765 (198)	ng eq./mL·h	10,300 (1970)	9,670 (1800)
*t*_1/2_	h	3.31 (0.408)	3.21 (0.534)	2.94 (0.259)	h	4.83 (1.63)	8.60 (2.67)
*T*_max_ ^a^	h	0.417 (0.33, 0.5)	0.75 (0.5, 1.5)	0.875 (0.5, 1)	h	0.75 (0.5, 2)	0.5 (0.5, 2)
CL/F	L/h	58.5 (14.6)	—	—	L/h	10.0 (1.92)	10.7 (2.18)
V_Z_/F	L	286 (105)	—	—	L	66.5 (10.3)	126 (19.7)

^a^ The table reports the median (minimum and maximum).

CL/F, apparent clearance; V_Z_/F, apparent volume of distribution; -, not detected.

LC-MS/MS quantification of SPH3127 in plasma. The mean plasma concentration-time profile of the parent drug SPH3127 is shown in Figure 3A, and its PK parameters are presented in Table 1. The arithmetic mean C_max_ and ${\text{AUC}}_{0-\infty }$ were 691 ng/mL and 1790 ng/mL·h, respectively. The median T_max_ and arithmetic mean t_1/2_ were 0.417 and 3.31 h, respectively.

LC-MS/MS quantification of M7-4 in plasma. The mean plasma concentration-time profile of the metabolite M7-4 is shown in Figure 3A, and its PK parameters are presented in Table 1. The arithmetic mean C_max_ and ${\text{AUC}}_{0-\infty }$ were 526 ng/mL and 2,220 ng/mL·h, respectively. The median T_max_ and arithmetic mean t_1/2_ were 0.75 h and 3.21 h, respectively.

LC-MS/MS quantification of M8-7 in plasma. The mean plasma concentration-time profile of the metabolite M8-7 is shown in Figure 3A, and its PK parameters are presented in Table 1. The arithmetic mean C_max_ and ${\text{AUC}}_{0-\infty }$ were 219 ng/mL and 765 ng/mL·h, respectively. The median T_max_ and arithmetic mean t_1/2_ were 0.875 h and 2.94 h, respectively.

The C_max_ and AUC values of SPH3127 were highest, followed by M7-4, with M8-7 the lowest, which was consistent with the proportions of various compounds in plasma.

### 3.3 Mass balance

In the six healthy Chinese male subjects, the mean total radioactivity recovery was 101.66% (ranging from 94.34% to 105.24%) within 192 h after the oral administration of 100 mg of [^14^C]SPH3127 (100 µCi). Both fecal and urinary excretion routes were the primary elimination routes, accounting for 57.31% and 44.35% of the administered dose, respectively. The cumulative recovery of radioactivity in urine and feces was depicted in Figure 3B.

### 3.4 HR-MS analysis of SPH3127 and reference standards of two possible metabolites

HR-MS was performed on a solution of reference standards of SPH3127, M7-4, and M8-7 to determine their mass fragmentation patterns and chromatographic behaviours, which were used to determine potential metabolites.

**FIGURE 4:**
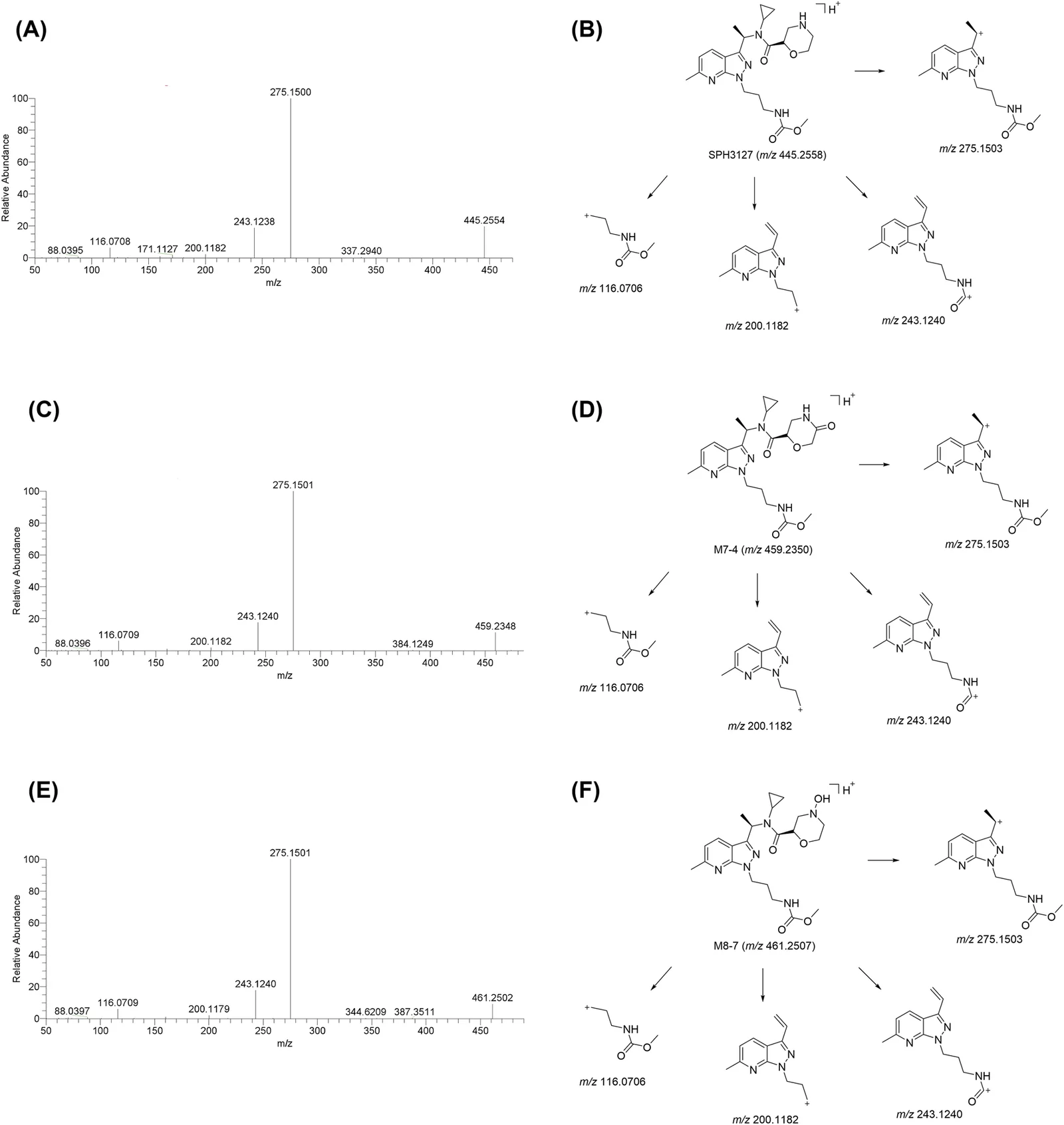
Product ion spectra and proposed fragmentation patterns of reference substances. SPH3127 **(A,B)**, M7-4 **(C,D)**, M8-7 **(E,F)**.

SPH3127, C_22_H_32_N_6_O_4_, with [M + H]^+^ at m/z 445.2558, eluted at 28.70 min and showed product ions at m/z 275.1503, 243.1240, 200.1182, and 116.0706 (Figures 4A,B). The base peak ion at m/z 275.1503 was generated by N-C cleavage.

M7-4, C_22_H_30_N_6_O_5_, with [M + H]^+^ at m/z 459.2350, eluted at 35.06 min and showed product ions at m/z 275.1503, 243.1240, 200.1182, and 116.0706 (Figures 4C,D). The base peak ion at m/z 275.1503 was generated by N-C cleavage. M7-4 was generated from SPH3127 by mono-oxidation on the carbon atom adjacent to the nitrogen atom of the morpholine.

M8-7, C_22_H_32_N_6_O_5_, with [M + H]^+^ at m/z 461.2507, eluted at 33.48 min and showed product ions at m/z 275.1503, 243.1240, 200.1182, and 116.0706 (Figures 4E,F). The base peak ion at m/z 275.1503 was generated by N-C cleavage. M8-7 was generated from SPH3127 by mono-oxidation on the nitrogen atom of the morpholine.

### 3.5 Metabolite profiling and identification

**FIGURE 5:**
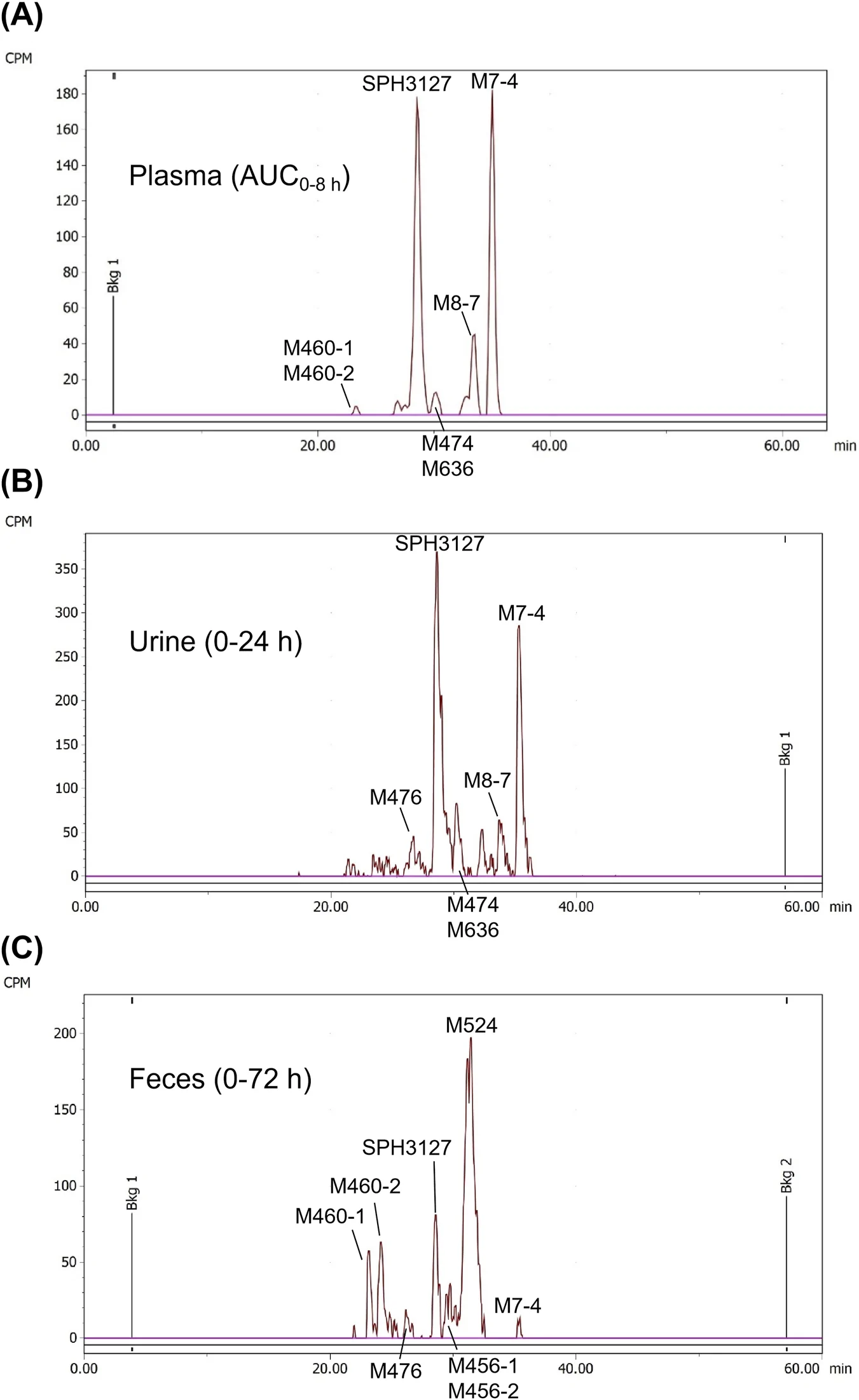
Representative radio-chromatograms of metabolites in human plasma (0–8 h, **(A)**, urine (0–24 h, **(B)**, and feces (0–72 h, **(C)** following oral administration of 100 mg [^14^C]SPH3127 (100 μCi).

Radio-chromatograms of each matrix are shown in Figure 5. Table 2 summarizes some characteristics of 12 metabolites, from which the structure identification of metabolites can also be confirmed. The percent values of SPH3127 and its major metabolites in pooled plasma, urine, and feces are listed in Table 3. The nomenclature for the newly identified metabolites was established by prefixing “M” to the molecular weight of each compound, except for the reference standards.

**TABLE 2:** Information of SPH3127 metabolites detected in human plasma, urine, and feces by using UHPLC-Q exactive plus MS.

ID	Metabolic pathway	Formula	Ring and double Bond equivalents	[M + H]^+^ *m/z* (theoretical)	[M + H]^+^ *m/z* (measured)	Mass error (ppm)	Fragment ions
SPH3127	Parent drug	C_22_H_32_N_6_O_4_	10	445.2558	445.2553	−1.1	275.1503, 243.1240, 200.1182, 116.0706
M401	Hydrolysis + oxidation	C_20_H_27_N_5_O_4_	10	402.2136	402.2132	−1.0	232.1081, 214.0975, 160.0869
M456-1	[O]+2[−2H]	C_22_H_28_N_6_O_5_	12	457.2194	457.2185	−2.0	344.1717, 273.1346, 160.0869
M456-2	[O]+2[−2H]	C_22_H_28_N_6_O_5_	12	457.2194	457.2187	−1.5	344.1717, 273.1346, 160.0869
M458-1	[O]+[−2H]	C_22_H_30_N_6_O_5_	11	459.2350	459.2330	−4.4	275.1503, 243.1240, 200.1182, 116.0706
M7-4	[O]+[−2H]	C_22_H_30_N_6_O_5_	11	459.2350	459.2345	−1.1	275.1503, 243.1240, 200.1182, 116.0706
M460-1	[O]	C_22_H_32_N_6_O_5_	10	461.2507	461.2498	−2.0	443.2401, 368.2081, 273.1346, 216.1131, 160.0869, 114.0550
M460-2	[O]	C_22_H_32_N_6_O_5_	10	461.2507	461.2498	−2.0	443.2401, 368.2081, 273.1346, 216.1131, 160.0869, 114.0550
M8-7	[O]	C_22_H_32_N_6_O_5_	10	461.2507	461.2502	−1.1	275.1503, 243.1240, 200.1182, 116.0706
M474	2[O]+[−2H]	C_22_H_30_N_6_O_6_	11	475.2300	475.2278	−4.6	457.2194, 358.1874, 275.1503, 243.1240, 116.0706
M476	2[O]	C_22_H_32_N_6_O_6_	10	477.2456	477.2448	−1.7	275.1503, 243.1240, 200.1182, 116.0706
M524	[SO_3_]	C_22_H_32_N_6_O_7_S	10	525.2126	525.2119	−1.3	445.2558, 275.1503, 243.1240, 116.0706
M636	[O]+[GluA]	C_28_H_40_N_6_O_11_	12	637.2828	637.2820	−1.3	461.2507, 275.1503, 243.1240, 116.0706

[GluA], glucuronidation; [O], oxidation; [SO_3_], sulfation; [-2H], dehydrogenation; [2H], reduction. The HR-MS, and HR-MS^2^, acquisition were performed on a Vanquish UHPLC, system coupled with a Q Exactive Plus mass spectrometer equipped with ESI, source.

**TABLE 3:** Percent distribution of SPH3127 and its metabolites in pooled plasma, urine and feces from human subjects.

ID	Retention time (min)	Plasma	Urine		Feces		Urine + Feces
			44.35% of dose*		57.31% of dose*		101.66% of dose*
		%AUC	%Urine	%Dose	%Feces	%Dose	%Dose
SPH3127	28.50–28.62	44.29	39.14	17.36	10.00	5.73	23.09
M460-1	23.02–23.40	1.10	2.39	1.06	6.66	3.82	10.28
M460-2	23.40–24.07				9.43	5.40	
M401	24.60	–	1.49	0.66	–	–	0.66
M476	26.35–26.95	–	6.35	2.82	3.26	1.87	4.69
M474	30.00–30.30	3.48	7.10	3.15	–	–	3.15
M636					–	–	
M456-1	29.70	–	–	–	7.80	4.47	4.47
M456-2		–	–	–			
M524	31.13	–	–	–	53.95	30.92	30.92
M458-1	32.83	2.39	1.31	0.58	–	–	0.58
M8-7	33.33–33.85	9.99	6.57	2.91	–	–	2.91
M7-4	35.00–35.28	35.53	22.13	9.81	1.81	1.04	10.85

– no prominent chromatographic peak was observed in the radioactive chromatogram.

* The percentage of total radioactivity from identified and unidentified substances relative to the administered dose.

%AUC, derived from 0–8 h AUC-pooled plasma; %Urine derived from 0–24 h pooled urine; %Feces derived from 0–72 h pooled feces; %Dose derived from the 0–192 h pooled urine and feces.

#### 3.5.1 Plasma

In human plasma, the radioactivity of SPH3127 and identified metabolites accounted for 96.78% of the total exposure; SPH3127 accounted for 44.29% of the total exposure. Combined with the HR-MS analysis, seven metabolites were detected in plasma, of which M7-4 was the most abundant metabolite, accounting for 35.53% of the total exposure; M8-7 was the second most abundant metabolite, accounting for 9.99% of the total exposure; other metabolites accounted for less than 4% of the total exposure (Figure 5A).

M7-4. The protonated molecular ion [M + H]^+^ of M7-4 was detected at m/z 459.2350, with a formula of C_22_H_30_N_6_O_5,_ based on exact mass measurement. The structure of M7-4 was confirmed by comparing the retention time and LC-MS/MS spectrum with those of the reference standard (Figures 4C,D).

M8-7. The protonated molecular ion [M + H]^+^ of M8-7 was detected at m/z 461.2507, with a formula of C_22_H_32_N_6_O_5_ based on exact mass measurement. The structure of M8-7 was confirmed by comparing the retention time and LC-MS/MS spectrum with those of the reference standard (Figures 4E,F).

Several metabolites (M460–1, M460–2, M474, M636, and M458-1) were identified as minor metabolites of SPH3127. M474 and M636 co-eluent, M458-1, and M460–1 and M460-2 co-eluent accounted for 3.48%, 2.39%, and 1.10% of the total plasma radioactivity, respectively (Table 3).

#### 3.5.2 Urine

The total radioactive components excreted in human urine accounted for 44.35% of the dose, and SPH3127 accounted for 17.36% of the dose. Combined with the HR-MS analysis, nine metabolites were detected, of which M7-4 was the most abundant metabolite, accounting for 9.81% of the dose; M474 and M636 co-eluent were the second most abundant metabolites, accounting for 3.15% of the dose (Figure 5B).

##### 3.5.2.1 M474

**FIGURE 6:**
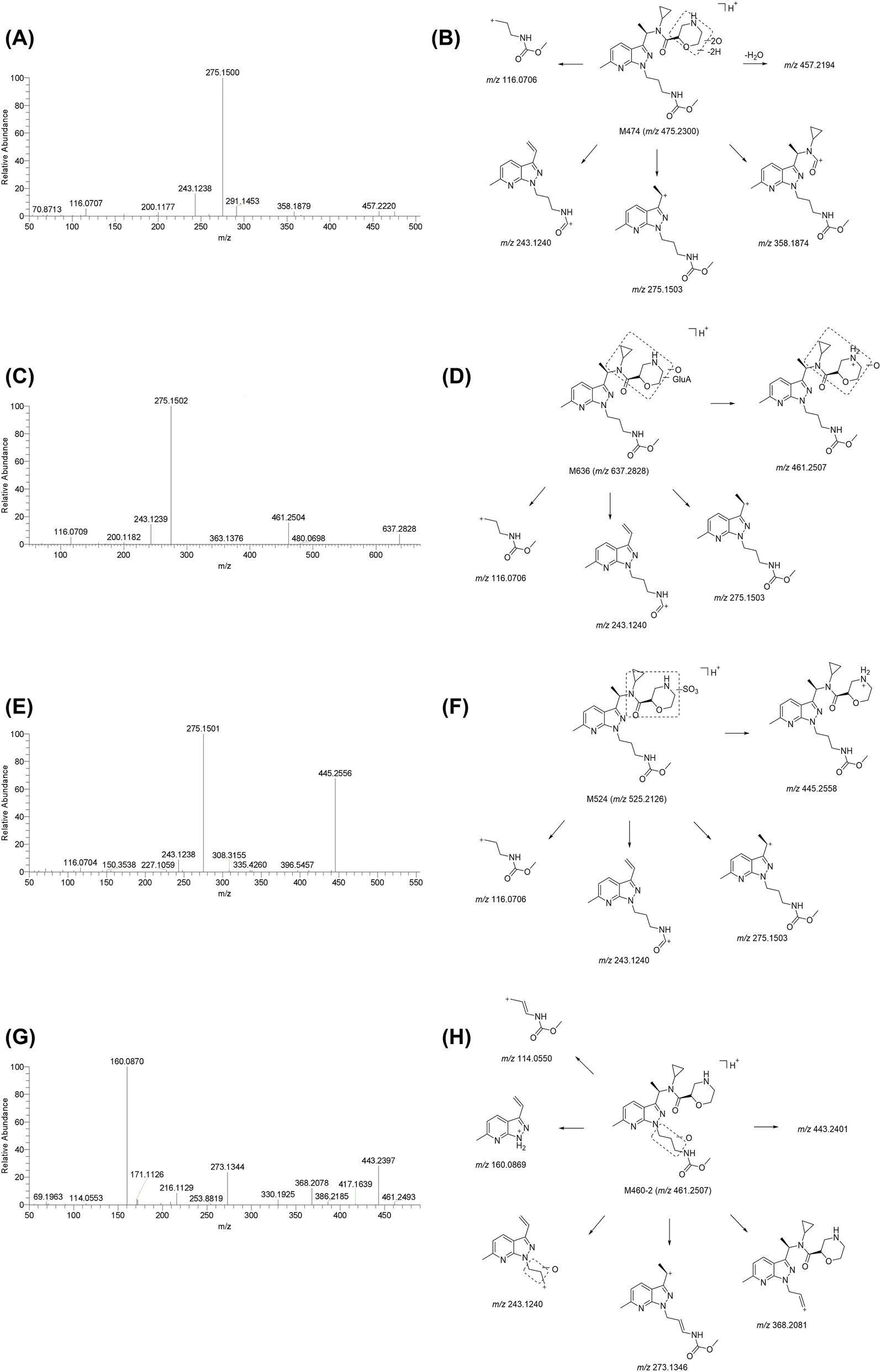
Product ion spectra and proposed fragmentation patterns of metabolites. M474 **(A,B)**, M636 **(C,D)**, M524 **(E,F)**, M460-2 **(G,H)**.

The retention time of M474 was 30.16 min, and its [M + H]^+^ was m/z 475.2300. The exact mass indicated molecular formula of C_22_H_30_N_6_O_6_, which was H_2_ less and two O atoms more than SPH3127 (C_22_H_32_N_6_O_4_), suggesting that M474 was derived from SPH3127 by dehydrogenation and dioxidation. The major product ion m/z 275.1503 corresponded to the molecular formula C_14_H_19_O_2_N_4_, identical to the ion produced from SPH3127. The structure of the fragment ion at m/z 358.1874 remained unchanged compared to this portion of the parent drug, but the modified morpholine group was lost due to bond cleavage. The fragment ion at m/z 457.2194 was obtained from the neutral loss of one molecule of water from the precursor ion. It is speculated that M474 was generated from SPH3127 by dehydrogenation and dioxidation on the morpholine (Figures 6A,B).

##### 3.5.2.2 M636

The retention time of M636 was 29.94 min, and its [M + H]^+^ was m/z 637.2828. The exact mass indicated the molecular formula of C_28_H_40_N_6_O_11_, which was SPH3127 (C_22_H_32_N_6_O_4_) with the addition of C_6_H_8_O_7_. The fragment ion at m/z 461.2507 was obtained from the loss of C_6_H_8_O_6_, and contained one additional O atom than the SPH3127. The fragment ions at m/z 275.1503, 243.1240, and 116.0706 were the same as those of SPH3127. It is speculated that M636 was generated from SPH3127 by mono-oxidation and glucuronidation (Figures 6C,D). The metabolic sites could not be confirmed using only MS data.

#### 3.5.3 Feces

The total radioactive components excreted in human feces accounted for 57.31% of the dose, and SPH3127 accounted for 5.73% of the dose. Combined with the HR-MS analysis, seven metabolites were detected, of which M524 was the most abundant metabolite, accounting for 30.92% of the dose; M460-2 was the second most abundant metabolite, accounting for 5.40% of the dose (Figure 5C).

##### 3.5.3.1 M524

The retention time of M524 was 30.50 min, and its [M + H]^+^ was m/z 525.2126. The exact mass indicated the molecular formula of C_22_H_32_N_6_O_7_S, which was SPH3127 (C_22_H_32_N_6_O_4_) with the addition of SO_3_. The fragment ion at m/z 445.2558 was obtained from the loss of SO_3._ The fragment ions at m/z 275.1503, 243.1240, and 116.0706 were the same as those of SPH3127.

It is speculated that M524 was generated from SPH3127 by sulfonation (Figures 6E,F).

##### 3.5.3.2 M460-2

The retention time of M460-2 was 24.18 min, and its [M + H]^+^ was m/z 461.2507. The exact mass indicated molecular formula of C_22_H_32_N_6_O_5_, which was one O atom more than SPH3127 (C_22_H_32_N_6_O_4_), suggesting that M460-2 was derived from SPH3127 by mono-oxidation. The fragment ions at *m/z* 273.1346 and 114.0550 both lost H_2_ compared to the fragment ions m/z 275.1503 and 116.0706 of SPH3127, respectively. The fragment ion at *m/z* 216.1131 contained one additional O atom compared to the fragment ion m/z 200.1182 of SPH3127. It is speculated that M460-2 was generated from SPH3127 by mono-oxidation on the fatty chain connecting the pyrazole to amide group (Figures 6G,H).

#### 3.5.4 Other metabolites

There are minor metabolites detected in plasma, urine, and feces.

##### 3.5.4.1 M401

**FIGURE 7:**
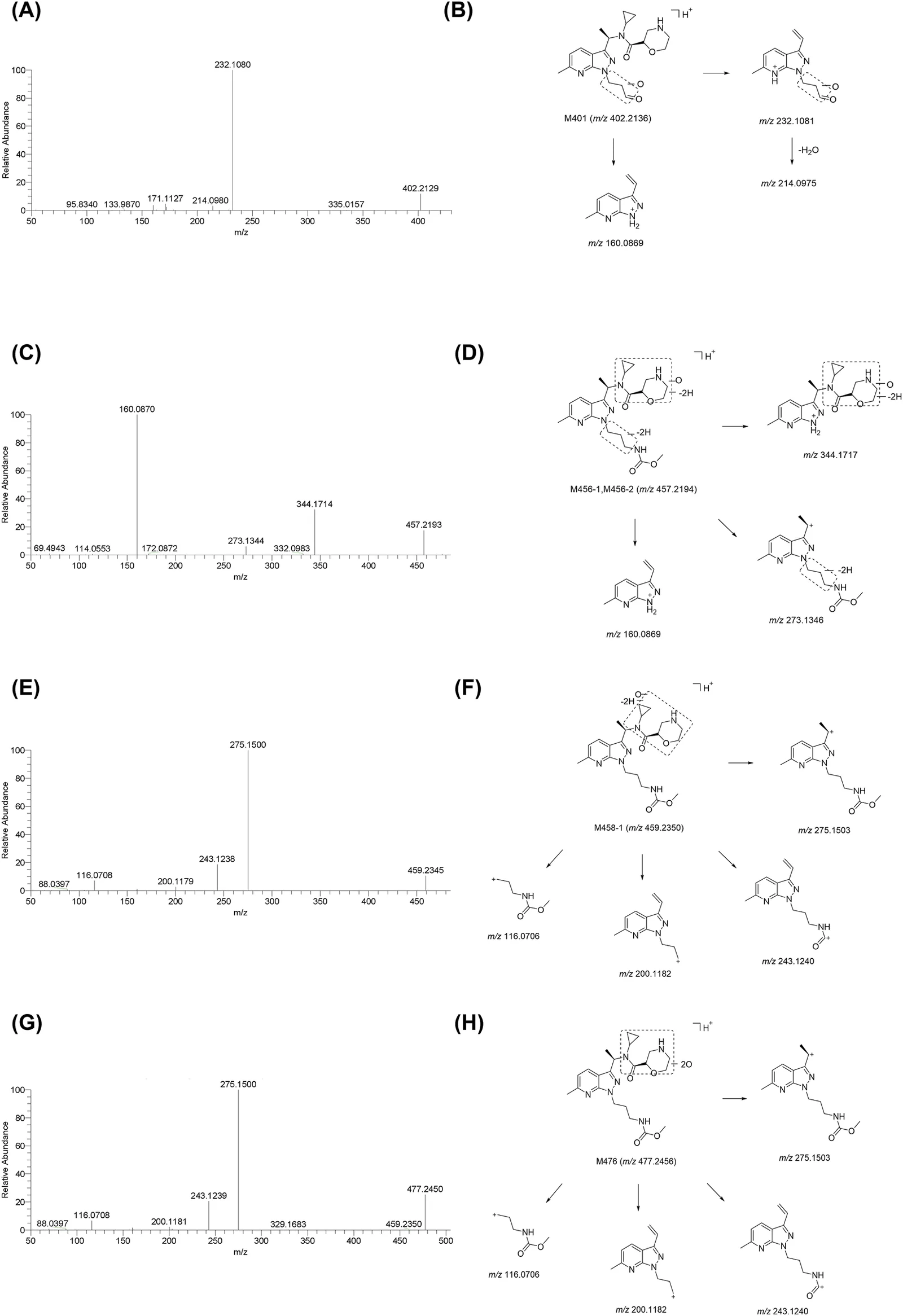
Product ion spectra and proposed fragmentation patterns of metabolites. M401 **(A,B)**, M456–1 and M456-2 **(C,D)**, M458-1 **(E, F)**, M476 **(G,H)**.

The retention time of M401 was 24.44 min, and its [M + H]^+^ was m/z 402.2136. M401, which has odd molecular weight, contains even numbers of nitrogen atoms in the protonated or deprotonated form. The exact mass indicated molecular formula of C_20_H_27_N_5_O_4_, which was 2 C atoms less, 5 H atoms less, and 1 N atom less than SPH3127 (C_22_H_32_N_6_O_4_), and the ring and double bond equivalents were the same as those of SPH3127, suggesting that M401 was derived from SPH3127 by amide hydrolysis and oxidation. The fragment ion at m/z 232.1081 indicated that the oxidation position of M401 was on the fatty chain connecting the pyrazole to amide group (Figures 7A,B).

##### 3.5.4.2 M456-1 and M456-2

The retention times of M456-1 and M456-2 were 28.33 min and 29.21 min, and their [M + H]^+^ was m/z 457.2194. The exact mass indicated molecular formula of C_22_H_28_N_6_O_5_, which was two pairs of H_2_ less and one O atom more than SPH3127 (C_22_H_32_N_6_O_4_), suggesting that M456-1 and M456-2 were derived from SPH3127 by dehydrogenation and oxidation. The fragment ion at m/z 273.1346 lost H_2_ compared to the fragment ion m/z 275.1503 of SPH3127. It is speculated that M456-1 and M456-2 were generated from M458-1 by dehydrogenation on the fatty chain connecting the pyrazole to amide group (Figures 7C,D).

##### 3.5.4.3 M458-1

The retention time of M458-1 was 35.08 min, and its [M + H]^+^ was m/z 459.2350. The exact mass indicated molecular formula of C_22_H_30_N_6_O_5_, which was H_2_ less and one O atom more than SPH3127 (C_22_H_32_N_6_O_4_), suggesting that M458-1 was derived from SPH3127 by dehydrogenation and oxidation. The fragment ions at m/z 275.1503, 243.1240, 200.1182, and 116.0706 were the same as those of SPH3127. It is speculated that M458-1 was generated from SPH3127 by dehydrogenation and oxidation on the morpholine (Figures 7E,F).

##### 3.5.4.4 M476

The retention time of M476 was 26.06 min, and its [M + H]^+^ was m/z 477.2456. The exact mass indicated molecular formula of C_22_H_32_N_6_O_6_, which was two O atoms more than SPH3127 (C_22_H_32_N_6_O_4_), suggesting that M476 was derived from SPH3127 by dioxidation. The fragment ions at m/z 275.1503, 243.1240, 200.1182 and 116.0706 were the same as those of SPH3127. It is speculated that M458-1 was generated from SPH3127 by dioxidation on the morpholine (Figures 7G,H).

**FIGURE 8:**
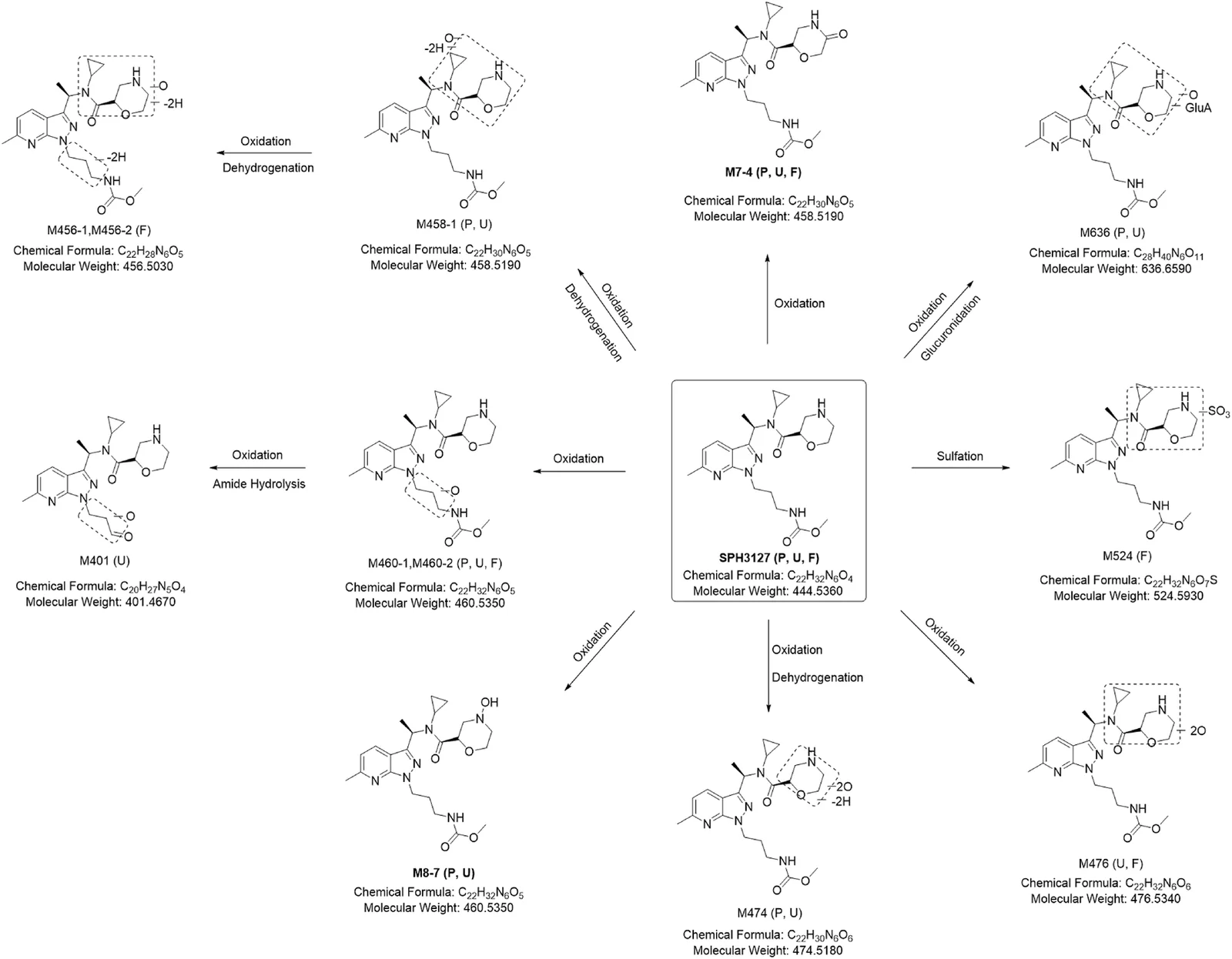
Metabolic pathway of SPH3127 in healthy Chinese male subjects (P, plasma; U, urine; F, feces).

The proposed metabolic pathway of SPH3127 in healthy Chinese male subjects is depicted in Figure 8.

## 4 Discussion

This study elucidates the pharmacokinetics, mass balance, and metabolism of [^14^C]SPH3127 in humans. Six healthy Chinese male subjects orally ingested 100 mg suspension of [^14^C]SPH3127 (100 µCi), and the metabolic characteristics of SPH3127 were investigated in human plasma, urine, and feces. According to the “Clinical Pharmacology Considerations for Human Radiolabeled Mass Balance Studies (2024)” from the FDA, the formulation used in the mass balance study contains both radiolabeled and non-radiolabeled drug materials. The formulation used in the study is appropriate for the study objectives and may differ from the final marketed product. Although formulation differences may cause some changes in ADME parameters (e.g., absorption), the formulation used in the study should still permit the collection of sufficient information to fulfill the objectives of the mass balance study.

After 192 h of dosing, 101.66% of the total drug-related component radioactivity was recovered in feces (57.31%) and urine (44.35%), indicating that the proportion of SPH3127 and related material excreted through urine and feces was similar and that SPH3127 was nearly completely excreted in humans. The total recovery was calculated based on the cumulative measurements of radioactivity in urine and feces. The slight deviation from 100% may result from the cumulative effect of uncertain factors, such as determination of the administered radioactive dose, measurement of sample weight, homogenization and aliquoting of feces, combustion efficiency, scintillation counting, background correction, and conversion of counts per minute to disintegrations per minute.

Based on the high radioactivity recovery, the UHPLC-HRMS analysis identified 12 metabolites of SPH3127 in human plasma, urine, and feces, 10 of which were phase I metabolites. The main metabolic pathways were oxidation, dehydrogenation, and amide hydrolysis. Additionally, 2 phase II metabolites were identified, including one glucuronide and one sulfated metabolite. The biotransformation pathway inferred from the results of the current metabolism study is depicted in Figure 8, and information on SPH3127 metabolites detected in human plasma, urine, and feces is provided in Table 3.

In human plasma, M7-4 was the most abundant metabolite, accounting for 35.53% of the total exposure to drug-related components. M8-7 was the second most abundant metabolite, accounting for 9.99% of the total exposure to drug-related components. M7-4 and M8-7 have the reference standards to verify their structures. The AUC-pooling method used in this study involves pooling appropriate aliquots of the plasma samples obtained from some time points, yielding one sample that has a concentration proportional to the pharmacokinetic AUC. This ensures that the pooled sample accurately reflects the average relative abundance of metabolites in the body. According to the “Safety Testing of Drug Metabolites (2020)” from the FDA, if the level of metabolites in humans is higher than 10% of the total drug exposure at a steady state, safety concerns are usually required. This study has confirmed the structures of M7-4 and M8-7; further investigation is needed to evaluate their safety.

In human urine, M7-4 was the most abundant metabolite, accounting for 9.81% of the dose. M474 and M636 co-eluent were the second most abundant metabolites, accounting for 3.15% of the dose. M636 was the glucuronidation product of SPH3127. Glucuronidation is the most common phase II conjugation involved in drug metabolism. Generally, glucuronidated metabolites have good water solubility, no biological activity, and are easily excreted from the body (Shulami et al., 1999). M474 was generated from SPH3127 by dehydrogenation and dioxidation on the morpholine. Because M474 and M636 flow together, their respective specific proportions cannot be obtained.

In human feces, M524 was the most abundant metabolite, accounting for 30.92% of the dose. M460-2 was the second most abundant metabolite, accounting for 5.40% of the dose. M524 was the sulfation product of SPH3127. According to the fragmentation spectrum of M524, the fragment ion at m/z 445.2558 generated from the loss of sulfuric acid (−79.9568 Da) was observed (Figure 6F), but we were unable to determine whether sulfuric acid was bound to the O or N atom. The structure of M524 remains unconfirmed due to the absence of a reference standard for verification. This study also showed that M524 was not detected in human plasma and urine. Many drugs are metabolized by the liver, particularly through conjugation reactions such as conjugation with glucuronic acid, sulfate, or glutathione. Following these reactions, the molecular weight of the metabolites increases significantly, and their polarity is greatly enhanced. These metabolites, characterized by their high molecular weight and high polarity, are likely to be substrates for transporters localized on the canalicular membrane of hepatocytes. It is possible that metabolites of SPH3127 are excreted into the feces via bile after being metabolized in the liver. Some studies have shown that gut microbiota modulates xenobiotic metabolism through direct and indirect mechanisms, altering the efficacy and toxicity of xenobiotics and drugs, and demonstrating metabolic capabilities comparable to those of any organ in the body, including the liver (Yang et al., 2025; Mikov, 1994; Boer et al., 2016). Gut microbiota is responsible for various metabolic reactions, including conjugation (Liu et al., 2024; Correia et al., 2020 ^).^ It is also conceivable that gut microbiota may contribute to the formation of sulfate conjugates. The high amount of M524 in feces validated the above two hypotheses. However, these hypotheses need further exploration and validation.

The clinical PK profile of SPH3127 has been characterized in healthy volunteers after treatment with single and multiple doses (Jing et al., 2021). PK data for single doses of SPH3127 (25–800 mg) in humans showed that the drug exhibits rapid absorption, short half-life, and dose-proportional pharmacokinetic characteristics (25–800 mg). After administration, the plasma renin activity could be decreased. A significant trend of renin inhibition was found in each dose group. The results indicate that SPH3127 had extensive metabolism in the human body, and a total of 51 metabolites were detected. In absorption, distribution, metabolism, and excretion (ADME) studies, the use of ^14^C-labeled drugs offers significant advantages over traditional unlabeled methods. ^14^C labeling method can trace all drug-related substances, including the parent drug and all its metabolites, by measuring total radioactivity in biological matrices, thereby providing a clear assessment of mass balance and overall systemic exposure. For SPH3127, the radioactive metabolite profile provides a clear picture of the number of metabolites and enables the detection and accurate quantification of metabolites in complex biological samples without being affected by mass spectrometry ionization efficiency or UV absorption, which greatly facilitates metabolite profiling and structural identification.

To better understand the PK characteristics of SPH3127, it was compared with the renin inhibitor aliskiren. The plasma PK parameters following a single dose of aliskiren (300 mg) in healthy Chinese subjects showed a geometric mean C_max_ of 223.4 ng/mL, ${\text{AUC}}_{0-\infty }$ of 1,365 ng/mL·h, t_1/2_ of 50.1 h, apparent clearance of 237 L/h, and apparent volume of distribution of 21,602 L/h. The median T_max_ of aliskiren and SPH3127 were 1.0 and 0.417 h, respectively (Vaidyanathan et al., 2007). SPH3127 was absorbed significantly faster. In addition, the t_1/2_ of SPH3127 (3.31 h) was shorter than that of aliskiren (50.1 h), suggesting a lower risk of systemic accumulation under repeated dosing regimens. Aliskiren is metabolized to a very limited extent. Approximately 81% of plasma radioactivity was accounted for by unchanged aliskiren, indicating very low exposure to metabolites. SPH3127 is extensively metabolized via multiple pathways, including oxidation, dehydrogenation, hydrolysis, glucuronidation, and sulfation. Also, for aliskiren, radioactivity was excreted almost completely via the biliary/fecal route, with only 0.6% of the radioactive dose recovered in urine. Unabsorbed aliskiren accounted for a large proportion of the dose recovered in feces in unchanged form, and the absorbed fraction of aliskiren can be estimated to approximately 5% of the dose. The urinary recovery of unchanged SPH3127 (17.36% of the administered dose) is substantially greater than that reported for aliskiren (0.4% of the dose) (Waldmeier et al., 2007). The above findings suggest that SPH3127 has a higher oral bioavailability compared with aliskiren.

This study was designed as a human radiolabeled mass-balance and metabolite profiling study. Its primary objectives were to characterize the excretion routes, overall recovery of administered radioactivity, and metabolic profiles of SPH3127 after a single oral dose, rather than to establish definitive population pharmacokinetics and evaluate sex-related differences. The enrollment of six healthy male subjects in this human radiolabeled mass balance study is consistent with guidance. In general, a mass balance study should include at least six evaluable volunteers. The decision to enroll only male subjects is also justified by both regulatory and practical considerations, which can reduce physiological variability during the initial characterization of human mass balance and metabolite disposition and avoid potential embryo-fetal radiation exposure associated with administration of a radiolabeled investigational product.

Sex-related differences in drug disposition may arise from differences in body composition, gastrointestinal physiology, plasma protein binding, renal function, and drug-metabolizing enzyme or transporter activity. Such effects are drug-specific and may be clinically relevant for some compounds. For example, a population pharmacokinetic analysis of levetiracetam found approximately 12% higher exposure in females than in males, whereas ethnicity was not a significant covariate after adjustment for other factors (Pigeolet et al., 2007). In contrast, a study of posaconazole in healthy volunteers found no clinically relevant effects of sex or race/ethnicity on pharmacokinetics (Sansone-Parsons A et al., 2007). Future studies, including female participants and more ethnically diverse populations, as well as population pharmacokinetic analyses, are needed to evaluate the clinical relevance of these factors.

Through radiolabeled mass balance studies in humans, drug-related radioactivity in plasma, urine, and feces can be quantitatively tracked, thereby enabling a comprehensive assessment of metabolic pathways and routes of elimination. Such information can identify major circulating and excreted metabolites for further safety considerations and provide a basis for the design of subsequent clinical pharmacology studies, including drug interactions, renal or hepatic impairment, and population pharmacokinetic studies. The PK characteristics of SPH3127 in patients with hepatic or renal impairment may change following administration of SPH3127. In patients with hepatic impairment, the proportion of the parent drug in the blood may increase. In patients with renal impairment, reduced glomerular filtration and tubular secretion could lead to accumulation of the parent drug and its metabolites, potentially influencing the overall exposure and safety profile. There is potential for renal impairment to also alter some drug metabolism and transport pathways in the liver and gut. The comprehensive PK and mass balance data from this study provide clinical guidance for the administration to patients with hepatic or renal impairment.

In conclusion, after oral administration of 100 mg (100 µCi) of [^14^C]SPH3127 to six healthy Chinese male volunteers, SPH3127 was quickly absorbed with the T_max_ of 0.417 h. Moreover, 101.66% of the administered dose of radiopharmaceutical-related components was excreted within 192 h (44.35% in urine and 57.31% in feces). In addition to the parent drug, M7-4 was the most abundant metabolite detected in human plasma and urine. M524 was the most abundant metabolite detected in human feces.

The comprehensive pharmacokinetic characterisation and mass balance analysis provide critical insights into the metabolic pathway and elimination mechanisms of SPH3127, and help prescribers to adjust the dose in patients with liver or kidney damage.

## Data Availability

The original contributions presented in the study are included in the article/Supplementary Material, further inquiries can be directed to the corresponding authors.
