# Supplementary material for: Pharmacokinetics, mass balance, and metabolism of [14C]SPH3127 (sitokiren), a novel direct renin inhibitor in humans
Source: Front Pharmacol. 2026 Sep 1;17:1893643. doi: 10.3389/fphar.2026.1893643 (PMC13574930; doi:10.3389/fphar.2026.1893643)
Supplement: Supplementary file 1 [file Table1.docx]

**Supplemental** **tables**

TABLE 1 Intra batch and Inter batch precision and accuracy of SPH3127, M7-4 and M8-7.

| Compound | Matrix | | n | LLQC | | | LQC | | | MQC | | | HQC | | |
| --- | --- | --- | --- | --- | --- | --- | --- | --- | --- | --- | --- | --- | --- | --- | --- |
|  | Human plasma | |  | Mean | RE (%) | CV (%) | Mean | RE (%) | CV (%) | Mean | RE (%) | CV (%) | Mean | RE (%) | CV (%) |
| SPH3127 | Intra batch | Batch1 | 6 | 1.03 | 3.1 | 6.5 | 3.32 | 10.7 | 2.6 | 54.8 | 9.6 | 0.9 | 840 | 5.0 | 1.8 |
|  |  | Batch2 | 6 | 1.07 | 7.3 | 2.7 | 2.99 | -0.4 | 3.0 | 54.8 | 9.6 | 2.4 | 806 | 0.7 | 0.8 |
|  |  | Batch3 | 6 | 1.03 | 3.0 | 4.6 | 3.07 | 2.3 | 1.8 | 54.9 | 9.8 | 2.6 | 827 | 3.4 | 3.0 |
|  | Inter batch |  | 18 | 1.04 | 4.5 | 5.0 | 3.13 | 4.2 | 5.2 | 54.9 | 9.7 | 2.0 | 824 | 3.0 | 2.6 |
| M7-4 | Intra batch | Batch1 | 6 | 1.94 | -3.0 | 4.5 | 6.64 | 10.7 | 4.2 | 113 | 12.7 | 1.2 | 1723 | 7.7 | 2.2 |
|  |  | Batch2 | 6 | 2.13 | 6.3 | 4.7 | 5.99 | -0.2 | 2.8 | 110 | 10 | 3.0 | 1620 | 1.3 | 0.7 |
|  |  | Batch3 | 6 | 2.11 | 5.7 | 4.0 | 6.03 | 0.5 | 1.8 | 110 | 9.5 | 2.6 | 1633 | 2.1 | 3.1 |
|  | Inter batch |  | 18 | 2.06 | 3.0 | 5.9 | 6.22 | 3.7 | 5.7 | 111 | 10.7 | 2.6 | 1659 | 3.7 | 3.5 |
| M8-7 | Intra batch | Batch1 | 6 | 0.409 | 2.2 | 16.9 | 1.34 | 11.8 | 8.4 | 21.9 | 9.4 | 1.1 | 337 | 5.2 | 1.8 |
|  |  | Batch2 | 6 | 0.403 | 0.6 | 15.5 | 1.17 | -2.2 | 5.4 | 21.9 | 9.6 | 2.5 | 329 | 2.9 | 0.6 |
|  |  | Batch3 | 6 | 0.380 | -5.1 | 11.1 | 1.21 | 0.8 | 3.3 | 21.7 | 8.4 | 3.1 | 330 | 3.1 | 3.1 |
|  | Inter batch |  | 18 | 0.40 | -0.7 | 14.3 | 1.24 | 3.5 | 8.4 | 21.8 | 9.1 | 2.3 | 332 | 3.8 | 2.2 |

For SPH3127, LLQC was 1.00 ng/mL; LQC was 3.00 ng/mL; MQC was 50.0 ng/mL; HQC was 800 ng/mL. For M7-4, LLQC was 2.00 ng/mL; LQC was 6.00 ng/mL; MQC was 100 ng/mL; HQC was 1600 ng/mL. For M8-7, LLQC was 0.40 ng/mL; LQC was 1.20 ng/mL; MQC was 20.0 ng/mL; HQC was 320 ng/mL.

TABLE 2 The matrix effects of SPH3127, M7-4, and M8-7 in hemolytic plasma and lipemic plasma.

| Matrix | n | SPH3127 | | | | | | | M7-4 | | | | | | M8-7 | | | | | |
| --- | --- | --- | --- | --- | --- | --- | --- | --- | --- | --- | --- | --- | --- | --- | --- | --- | --- | --- | --- | --- |
|  |  | LQC (3.00 ng/mL) | | | | HQC (800 ng/mL) | | | LQC (6.00 ng/mL) | | | HQC (1600 ng/mL) | | | LQC (1.20 ng/mL) | | | HQC (320 ng/mL) | | |
|  |  | Mean | | RE  (%) | CV  (%) | Mean | RE  (%) | CV  (%) | Mean | RE  (%) | CV  (%) | Mean | RE  (%) | CV  (%) | Mean | RE (%) | CV  (%) | Mean | RE (%) | CV (%) |
| Hemolytic plasma | 6 | 3.08 | 2.6 | | 2.6 | 848 | 6.0 | 1.7 | 6.18 | 2.9 | 1.8 | 1683 | 5.2 | 1.8 | 1.10 | -8.8 | 6.1 | 322 | 0.7 | 2.6 |
| Lipemic plasma | 6 | 3.00 | 0.1 | | 3.0 | 851 | 6.4 | 0.8 | 6.10 | 1.7 | 3.1 | 1718 | 7.4 | 1.0 | 1.11 | -7.5 | 7.4 | 351 | 9.7 | 0.5 |
